# Correction: Retention in care among people living with HIV in the national antiretroviral therapy programme in Guinea: A retrospective cohort analysis

**DOI:** 10.1371/journal.pgph.0002456

**Published:** 2023-09-28

**Authors:** Kadio Jean-Jacques Olivier Kadio, Amadou Cissé, Thierno Saidou Diallo, Foromo Guilavogui, Adrien Fapeingou Tounkara, Damey Pe, Alhassane Sow, Fatoumata Fily Bah, Souleymane Sékou Youla, Ibrahima Diallo, Niouma Nestor Leno, Lazare Mboungou, Nyawotope Koffi Arnold Ahiatsi, Laye Kaba, Sy Zeynabou, Ignasi Vallès-Casanova, Alison Wringe, Sarah Hoibak, Youssouf Koïta, Xavier Vallès

Multiple authors’ names are spelled incorrectly. The correct author names are: Kadio Jean-Jacques Olivier Kadio, Amadou Cissé, Thierno Saidou Diallo, Foromo Guilavogui, Adrien Fapeingou Tounkara, Damey Pe, Alhassane Sow, Fatoumata Fily Bah, Souleymane Sékou Youla, Ibrahima Diallo, Niouma Nestor Leno, Lazare Mboungou, Nyawotope Koffi Arnold Ahiatsi, Laye Kaba, Sy Zeynabou, Ignasi Vallès-Casanova, Alison Wringe, Sarah Hoibak, Youssouf Koïta, Xavier Vallès. The correct citation is: Kadio KJ-JO, Cissé A, Diallo TS, Guilavogui F, Tounkara AF, Pe D, et al. (2023) Retention in care among people living with HIV in the national antiretroviral therapy programme in Guinea: A retrospective cohort analysis. PLOS Glob Public Health 3(5): e0000970. https://doi.org/10.1371/journal.pgph.0000970

There are also errors in the Funding information. The correct Funding information is: This work was funded by the Global Fund to fight against AIDS, Malaria and Tuberculosis in the form of a grant (GIN-H-MOH) awarded to KJJOK, AC, TSD, FG, AFT, DPD, AS, FFB, SSY, ID, NNL, LM, NKAA, LK and YK. This work was also funded by Instiuto de Salud Carlos III, Ministry of Science and Innovation (Spain) through the study CLIMATE-COVID19 in the form of a grant (PIM-E-202030E222) awarded to IVC. The funders had no role in study design, data collection and analysis, decision to publish, or preparation of the manuscript.
