# Supplementary figures and images for: Retention in care among people living with HIV in the national antiretroviral therapy programme in Guinea: A retrospective cohort analysis
Source: PLOS Glob Public Health. 2023 May 16;3(5):e0000970. doi: 10.1371/journal.pgph.0000970 (PMC10187925; doi:10.1371/journal.pgph.0000970)

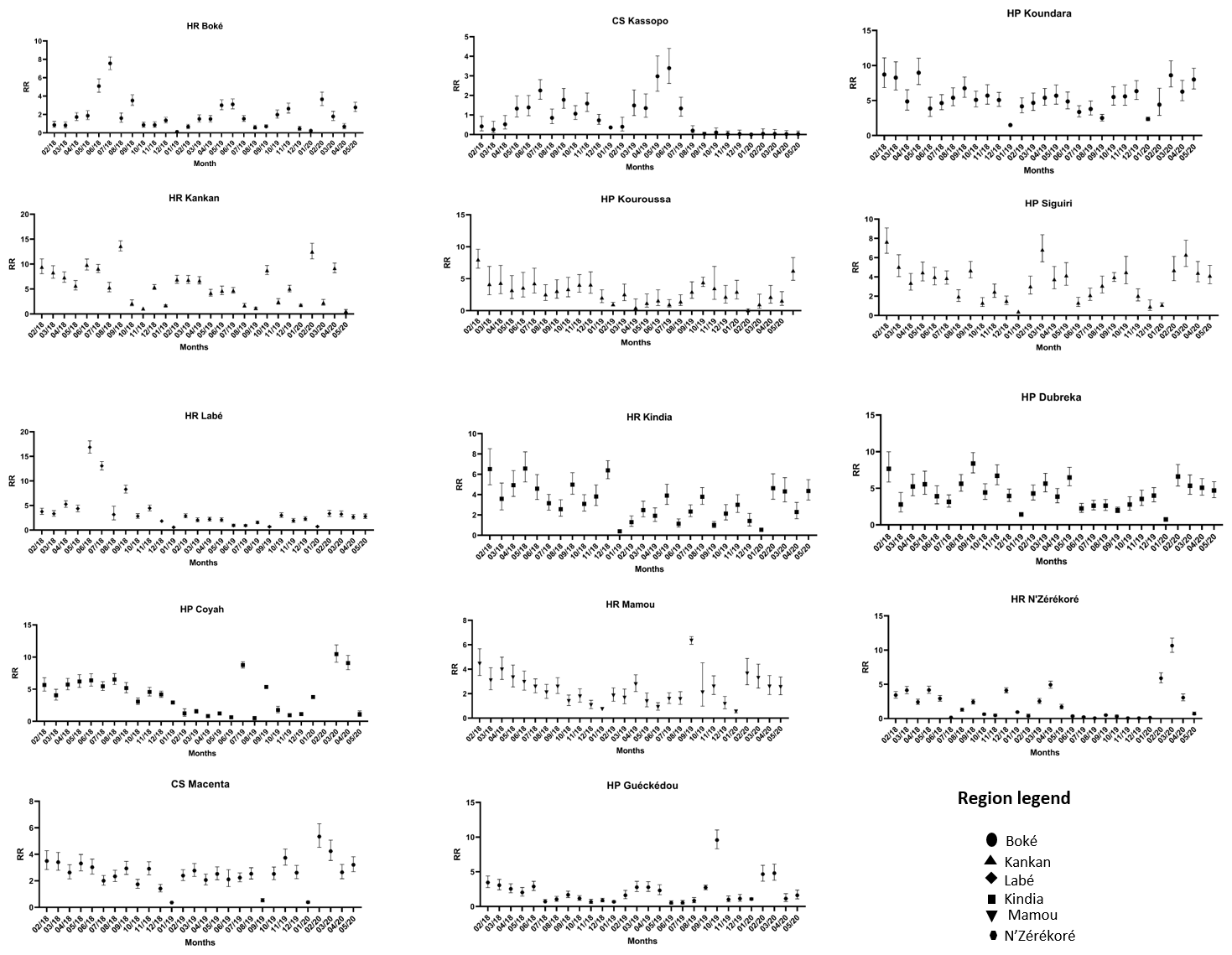

Supplement: S1 Fig — 1We included facilities with the larger cohorts (>300 patients) from each patients. Faranah region was excluded due the low numbers included in each site. (TIFF) [file pgph.0000970.s001.tiff]
