# Supplementary material for: Retention in care among people living with HIV in the national antiretroviral therapy programme in Guinea: A retrospective cohort analysis
Source: PLOS Glob Public Health. 2023 May 16;3(5):e0000970. doi: 10.1371/journal.pgph.0000970 (PMC10187925; doi:10.1371/journal.pgph.0000970)
Supplement: Attachment — Submitted filename: Answer_reviewers.docx [file pgph.0000970.s003.docx]

**Please use the space provided to explain your answers to the questions above. You may also include additional comments for the author, including concerns about dual publication, research ethics, or publication ethics. (Please upload your review as an attachment if it exceeds 20,000 characters)**

Reviewer #1: Peer-review report of the manuscript “Retention in care among people living with HIV in the national antiretroviral therapy programme in Guinea: a retrospective cohort analysis” (PGPH-D-22-01123).

Kadio and colleagues did a retrospective cohort study of rates and predictors of loss to follow-up and re-engagement in Guinea’s national ART program. The study reports concerningly low retention rates. The study also assessed associations between rainfall patterns and missed clinic visits and showed interesting correlations.

**Comment 1:** The manuscript is overall well written. Statistical analyses of rates and predictors of loss to follow-up and re-engagement are sound. However, analyses of associations between rainfall patterns and missed visits could be improved.

The most important limitation of the study is that the authors did not adjust loss to follow-up rates for undocumented mortality and silent transfer. Therefore, rates of loss to follow-up are likely to be substantially overestimated. I suggest the authors consider the likely overestimation of loss to follow-up rates in their conclusions.

***Answer:*** *We agree – our estimates of LTFU are likely to be over-estimated because they do not account for people who have moved between clinics without documentation and those who have died. Estimating the true number of LTFU, after adjusting for undocumented mortality and silent transfer is challenging in this setting as there are limited data sources from which the adjustment factors could be ascertained. This limitation is common across many ART cohort studies, and is one reason why our primary outcome is the proportion of patients who are retained in care.*

*Other published studies from sub-Sharan Africa have indicated that over 50% of patients who are designated as LTFU have either died or transferred elsewhere, without the transfer between clinics being documented (e.g reference 10). We have now referenced this point under the limitations. In the Discussion section (page 25, lines 444 to 455), we have additionally mentioned the need to establish clinic transfer procedures that allow movement between clinics for mobile patients to be better documented, which would improve the accuracy of LTFU estimates. See Discussion section, pages 24-25, lines 426-443:*

*“There are various limitations that need to be taken into account when interpreting our findings. Firstly, as in other countries with weak tracing and vital registration systems, the proportion of patients LTFU who had in fact self-transferred to another clinic or died could not be determined in our study. Our LTFU rate is likely over-estimated as studies from other contexts at a similar stage of scaling up ART have estimated that over 20% of patients LTFU patients had in fact died [10]. Furthermore, undocumented transfers to another clinic lead to an over-estimate of the number of PLHIV who have ever initiated ART, and under-estimates of retention in care rates.”*

*In light of these issues, we recommend strengthening the patient tracing system for patients who are LTFU, as well as implementing procedures to document transfers between clinics, such as the use of transfer forms that include unique patient identifiers. (Page 24-25, lines 438-443):*

*“Efforts to strengthen the tracing system, including follow up calls and visits once scheduled appointments are missed, would improve retention and also enable more accurate estimates of the effectiveness of the ART programme to be generated. Furthermore, implementing procedures to facilitate inter-clinic transfers would also improve the accuracy of estimates of the number of PLHIV who are LTFU”*

**Comment 2:** I have the following specific suggestions for improvement:

1. Analyses of rates and predictors of loss to follow-up and re-engagement:
a. Please clarify in the Methods section how you adjusted Cox models for the clustering of patients in facilities. Did you use cluster-based robust standard errors?

**Answer**: *We use cluster-based robust standard errors. We included this sentence in the Material and Methods section, page 10, line 206-207: “Cox models were adjusted for clustering of patients in facilities using cluster-based robust standard errors.”*

b. Adjusted model A could be removed from Tables 2 and 3. Analysis should be adjusted for the clustering of patients in facilities. Model A (adjusted for patient characteristics but not for clustering) is unnecessary.

**Answer:** *Thank you for your suggestion. We removed Model A from the tables 2 and 3.*

c. Please confirm the correctness of the results in Tables 2 and 3. If robust standard errors were used to adjust for clustering, I would expect that additional adjustment for clustering does not affect the point estimates but widens the confidence intervals. This is also the case for some but not for all estimates. For example, in Table 2, the adjusted hazard ratio for ART initiation for the year 2008 in model A is 2.10 (95 CI 2.00-2.20). In model B (additionally adjusted for clustering), the HR is the same as in model A, and the confidence interval is wider (aHR 2.10 95% CI 1.72-2.55). However, for patient characteristics (e.g. age and sex in Table 2), the point estimates and confidence intervals are identical in models A and B. Surprisingly, Table 3 point estimates for age, sex and out of Conakry are not identical in models A and B. Please double-check these results.

**Answer:** *Thank you for your observation. We double checked the results and confirm the results as has been shown in Table 2. These results should be interpreted as follows: some individual level characteristics like sex or age are not influenced by the site of follow up regarding the risk of LTFU. In other words, the quality of the service provided at site level does not discriminate between gender or age, and age and sex are individual independent variables regarding the risk of LTFU. We checked as well Table 3 and there were some mistakes in the adjustments estimates column. We introduced the necessary amendments highlighted in the tracked changes version, which do not affect the main findings of our article. We rephrased the following sentence which is more according to our results (Discussion section, pages 22-23, lines 383-390):*

*“The effect of adjusting for clustering at facility level regarding region, size and facility types suggests an important influence of the quality of the service provided in each setting. In contrast, the clustering effect was barely observed for sex and age (similar CI between when including or excluding clustering adjustments), which denotes that these individual-level factors are independent of the quality of service provided,(i.e. there is no discrimination over gender).”*

d. Conclusions regarding loss to follow-up rates should take into account likely overestimation due to lack of adjustment for silent transfer and undocumented mortality.

**Answer**: *We agree with your suggestion. See below the new version of the conclusions section (Page 26, lines 443-454):*

*“In conclusion, our study demonstrated high levels of LTFU and re-engagement in care, undermining the effectiveness and durability of first-line ART regimens. Tracing systems and clinic transfer procedures should be strengthened to more accurately ascertain the number of deaths and self-transfers which would enable the true number of persons LTFU to be determined. Nevertheless, even if LTFU rates are over-estimated, the rates of retention in care are sub-optimal in this setting as demonstrated by the high rate of re-engagement in care following treatment interruptions. Several studies have shown that multi-month dispensing and other differentiated service delivery models of ART [42] may improve care engagement, especially in rural areas [43,44] These interventions are slowly being introduced in Guinea, and subsequent evaluations of retention in care will be useful for further refining their application in this context. [45].”*

e. The data presented in the manuscript does not support the conclusion that tracing interventions, multi-month dispensing and other age and gender-specific interventions may improve care engagement. These interventions can be mentioned in the Discussion but should not be presented as Conclusions of this study.

**Answer:** *We have amended this point in the discussion. Please see our answer above.*

2. Analyses of associations between rainfall patterns and ART interruptions:
a. The analysis of rainfall patterns and ART interruptions is novel and topical and could be presented more prominently

**Answer:** *Thank you for your appreciation. A further study which aims to examine the interplay between climatic variables and health seeking behaviour is ongoing. However, to emphasize this aspect of the manuscript, we have now included some additional analysis and figures (see the following answer)*

b. The authors evaluated correlations between the average proportion of patients who missed a clinic visit outside of Conakry (5a) and in Conakry (5b) and the average rainfall in the entire country. Could this association be assessed on a facility level? I.e. the association between rainfall in the catchment area of a clinic and the proportion of patients who missed a clinic visit at this particular clinic.

**Answer:** *The national-level analysis suggests a substantial influence of rainfall patterns on retention in care, which is already presented and discussed. The analysis at a facility level is more difficult to interpret because other facility-level factors may influence retention including ART stock-outs, local cultural practices, migratory patterns and service quality. However, we included the following new figures to emphasize this analysis:*

1. *We have expanded figure 5 to add 5c which shows the Relative Risk of treatment interruption between in-country regions and Conakry region, by month (from February 2018 to May 2020)*
2. *We have included as supplementary material the same graphical analysis, but at facility level (Figure S1), including facilities with the higher cohorts from each region (except Faranah, from which the numbers were too small).*

*Accordingly, we included the following sentences in the Discussion, page 24, lines 424-429):*

*“Analyses of the association between rainfall in the catchment area of a clinic and the proportion of patients who missed a clinic visit at this particular clinic were more complicated to interpret because other unassessed factors could explain these differences including local-level ARV stock-outs, cultural practices, migratory patterns and service quality, some of which are also linked to climatic patterns.”*

c. Why are the peaks in missed visits in January 2019 and 2020 in panel 5a marked with a dashed line? Please clarify in the figure caption.

**Answer**: *We have now used a continuous line to avoid any confusion.*

d. The authors calculated Pearson’s correlation coefficient (r) to assess associations between rainfall patterns and missed visits but did not evaluate the statistical uncertainty of this association. The authors could use more advanced statistical methods to calculate odds or risk ratios with 95% confidence intervals for associations between rainfall patterns and missed visits on the facility level. For example, case-crossover designs are widely used to estimate associations between time-varying environmental exposures and health outcomes from daily time series data (see https://bmcmedresmethodol.biomedcentral.com/articles/10.1186/1471-2288-14-122). Such an analysis might be beyond the scope of this study and could be presented in a separate manuscript.

**Answer:** *Thank you for your suggestion. See our previous considerations about this. With this regard, we have included the RR and confidence intervals displayed in Figure 5c and S1. We are working in a separate manuscript to analyse in more depth the correlation between environmental variables and patterns of retention in care.*

Minor:
3. In the Abstract, the authors state that “patients initiating treatment between January 2018 and December 2020 were included” but in the Methods “ patients were included in the analysis if they had ever initiated ART between January 2018 and September 2020”. Please correct the end of the eligibility period in the Abstract or the Methods section.

**Answer:** *Sorry for this mistake. We included patients who initiated treatment prior to September 2020. The follow up period ended on December 2020 (cohort follow-up closure). The abstract has been modified accordingly.*

4. Reference 10 is not a review but a cohort study

**Answer:** *Thank you for spotting this error. We have now corrected this to indicate the study design (i.e. a cohort study).*

5. Please correct the number of included sites: in the Methods section, you state data from 73 facilities were used, but in the Abstract and Figure 1, you state 66 facilities were included.

**Answer:** *Data from a total of 73 facilities were included in the analysis. We have now corrected this in the Abstract, in Figure 1 and in the text in the manuscript.*

6. Line 168: Were scheduled appointment dates recorded in the database? Please clarify.

**Answer:** *The scheduled appointment dates were recorded in the database, however we considered the date of the ART refill and the number of days provided to ascertain when a patient was late for their next visit, and defined LTFU as commencing 90 days after that date.*

7. The following sentence is unclear and should be revised: “Patients under differential treatment with frequency refills, each refill was accounted for 2 or more consecutive months of ARV refill as appropriate.”

**Answer*:*** *We rephrased the sentence as follows: “Multi-month refills were taken into account when considering the LTFU start date.”*

8. I cannot follow the argument presented in lines 389-395 “The notorious effect of adjusting for clustering at facility level…”). Please rewrite this section.

**Answer:** *We rephrased this sentence as follows:*

“*We found some evidence of clustering at the facility level in terms of region, size and facility type, suggesting some differences in the effectiveness of service delivery in relation to these factors. This denotes most probably an important influence of the quality of the service provided in each setting.”*
