# Supplementary material for: Retention in care among people living with HIV in the national antiretroviral therapy programme in Guinea: A retrospective cohort analysis
Source: PLOS Glob Public Health. 2023 May 16;3(5):e0000970. doi: 10.1371/journal.pgph.0000970 (PMC10187925; doi:10.1371/journal.pgph.0000970)
Supplement: Attachment — Submitted filename: Reviewer_answer.docx [file pgph.0000970.s004.docx]

Journal Requirements:

1. Please review your reference list to ensure that it is complete and correct. If you have cited papers that have been retracted, please include the rationale for doing so in the manuscript text, or remove these references and replace them with relevant current references. Any changes to the reference list should be mentioned in the rebuttal letter that accompanies your revised manuscript. If you need to cite a retracted article, indicate the article’s retracted status in the References list and also include a citation and full reference for the retraction notice.

**Answer: We revised the reference list which is now completed. We only included an additional reference (now reference 46) upon a comment made by one of the reviewers (see below): “Cresswell FV, Lamorde M. Implementation of long-acting antiretroviral therapy in low-income and middle-income countries. Curr Opin HIV AIDS. 2022;doi: 10.1097/COH.0000000000000732.”**

2. Please insert an Ethics Statement at the beginning of your Methods section, under a subheading 'Ethics Statement'. It must include:

a) (for human participants/donors) - A statement that formal consent was obtained (must state whether verbal/written) OR the reason consent was not obtained (e.g. anonymity). NOTE: If child participants, the statement must declare that formal consent was obtained from the parent/guardian.

**Answer: The following statement have been introduced in the Ethical issues section (lines 232-233): “Consent to participants was waived by the Ethical Board due to the anonymity of the data sets used during the analysis.”**

Reviewer #2: The primary purpose of the paper was to fully document poor rates of retention in care among ART patients in Guinea’s national HIV treatment program with exploration as to possible causes of poor treatment retention through analysis of patient demographics, clinic specific factors, and a unique look at rainfall and other season agricultural factors that might influence ART continuation. The authors did an admirable effort to document the poor ART retention and included in the limitations section of the manuscript the issue of lack of records of patient mortality as well as lack of records regarding patient’s receiving ART care in other facilities other than the numerous clinics included in this study. Documentation of ART adherence and clinic retention is an important area of evaluation and research, particularly for those nations that fall below continental averages for ART adherence, which was the situation for Guinea.
The literature review was focused on the issues the current paper wished to address and established that retention in HIV care/ART adherence is a challenge for multiple medical care systems. The authors pointed out that the retention rates in Guinea at the clinics in question was lower than elsewhere in Africa, including in neighboring Mali. The literature review in the introduction set the stage for the problem faced by medical providers in Guinea.
Data collection and analysis was well documented and supported the claim of low ART adherence rates among multiple clinical providers. There was analysis that indicated the low ART adherence rate was higher among rural smaller clinics than larger urban clinics. But overall, the point was well established in their analysis that low ART adherence is a problem. There appeared to be no deviations from the proposed data collection and analysis plan. The authors point out that the rainfall data did not have the specificity for definitive analysis but was adequate for the conclusions they derived regarding ART adherence.
The paper is suitable for publication but there are a few typographical errors that need to be addressed. These include changing the word ‘African’ to ‘Africa’ in line 52; the correct word is ‘amalgamation’ in line 111; a comma was used instead of a decimal mark so that ‘1,7’ should be changed to ‘1.7’ on line 145; ‘rug-resistant’ should be changed to ‘drug-resistant on line 360; on line 373 consider whether ‘regionals’ or ‘regional’ is the best word to use; and online 420 the word ‘psychosocial’ is the correct term.

**Answer: These corrections have been introduced as indicated by the reviewer.**

The primary concern of this reviewer is with the final recommendations of multi-month ART dispersing and targeted ART adherence strategies for men and for younger HIV positive persons. The authors clearly state the problems with treatment failure and treatment resistance that may take place with sporadic ART adherence. However, their recommendations are not complete based upon the current science of ART adherence. Injectable ART for 30 days should have been considered in the conclusion section. Whereas multi-month ART dispersing would certainly be a sound recommendation, emerging research on 30-day and potentially 60-day ART injections may offer those HIV patients ART treatment who cannot travel during rainy season or while significant agricultural labors are required. This reviewer recommends a re-submission where the authors explore additional potential solutions to poor ART retention other than multi-month dispensing and ART adherence strategies designed for men and/or for younger HIV patients.

**Answer: Thank you for your suggestion. The following paragraph have been added at the end of the discussion section, lines 456-460: “A promising strategy would be the use of long-acting injectable ART which may reduce HIV treatment barriers with monthly or bi-monthly administration. However, evidence about feasibility and implementation considerations in LMICs is still lacking [46].” To support this statement we included the new reference [46] “Cresswell FV, Lamorde M. Implementation of long-acting antiretroviral therapy in low-income and middle-income countries. Curr Opin HIV AIDS. 2022;doi: 10.1097/COH.0000000000000732.”.**

The authors recommend further study of the effects of regional differences and climate factors on ART adherence. This would be important when implementing 30-day injectable ART since predicting the beginning of the rainy season would be essential to initiating a 30-day ART injection. The paper referred to the ability of those in Guiana to make predictions as to when the rainy season will begin.

**Answer: We fully agree with your comment, but at this stage, we consider too premature to speculate about use of injectable ART at an appropriate season, given the uncertainties which still remain about the feasibility/utility of injectable ART in real-world conditions.**

Data collection, cleaning, and analysis details in the methodology section was sufficient to allow others to reproduce this evaluation process with other clinical providers. The authors correctly note that improved tracing systems and clinic transfer procedures would strengthen the number of deaths and self-transfers so that a more accurate assessment of those lost to follow-up could be established. This is also a sound recommendation.
In summary, the paper is of high quality, well organized and clearly written, with only minor edits required. However, the inclusion of the potential of injectable ART as a final recommendation should be made prior to publication so that the paper is most current with the treatment literature.

Reviewer #3: This is a well-written and useful manuscript, thank you.
I have a few minor comments:
1. Although it is mentioned in the limitations, it is not clear in the methods that visits are ascertained at site level only and not across the country. Please explain in the methods that if a patient moves to another facility without documentation, that will be considered LTFU. And whether the unique identifier applies across regions?

**Answer: We included the following statement in the methods section, lines 169-172: “Visits are ascertained at site level and each patient has an unique identifier upon enrolement, but a new identifier was generated if a patient moves to another site without documentation or self-identification as PLVIH under ART.”**

Line 100- typo: disclosure- disclosure. The description of the setting is otherwise very helpful.
2. Line 112- would it not be more accurate to say “death or transfer not documented in clinic records”
3. Line 142 – typo: on- of
4. Table 1: typo 2020 column 19.8.4

**Answer: All these typos have been amended as indicated**

5. Figure5a- has a dashed line that is not explained: please explain

**Answer: This was a remaining of an old version of the figure. We resubmitted Figure 5a with a continuous line, now.**
